# Supplementary material for: Susceptibility of Diaphorina citri to Irradiation with UV-A and UV-B and the Applicability of the Bunsen–Roscoe Reciprocity Law
Source: Insects. 2023 May 8;14(5):445. doi: 10.3390/insects14050445 (PMC10231100; doi:10.3390/insects14050445)
Supplement: Supplementary file 1 [file insects-14-00445-s001.zip › insects-2346491-supplementary.pdf]

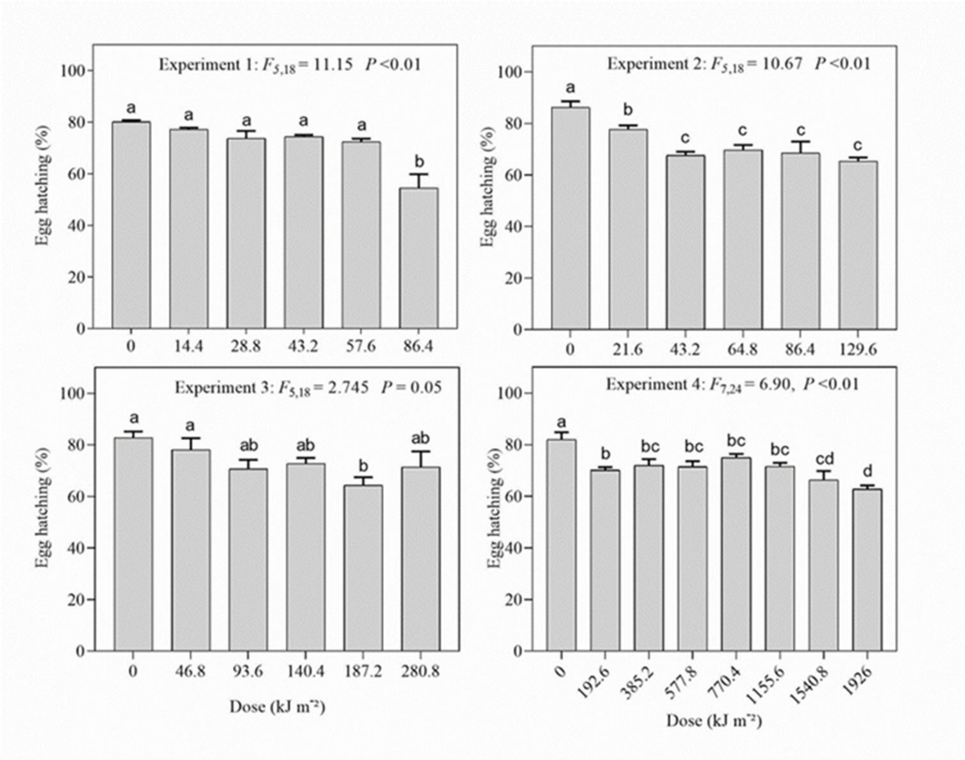

Figure S1. Effects of UV-A on the egg hatch of *D. citri*. In Expts. 1, 2, and 3, the eggs were exposed daily to 8, 12, and 26 W m^{-2} for 30 to 180 min and, in Expt. 4 they were exposed at 107 W m^{-2} for 30–300 min each day. The data are means (n = 4; 24–185 eggs per replication), and the bars represent the standard errors. Means annotated with different letters are significantly different from each other according to Duncan's MRT tests at p = 0.05.

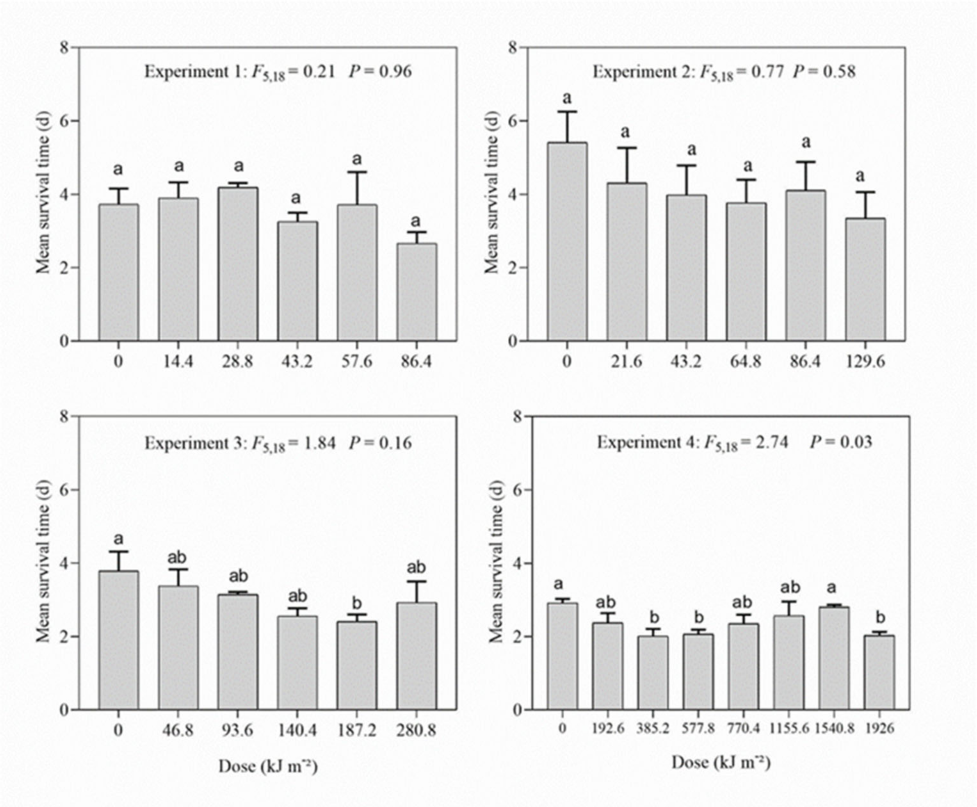

Figure S2. Effects of UV-A on survival of nymphs hatching from irradiated eggs of *D. citri.* In Expts. 1, 2, and 3, the eggs were exposed daily to 8, 12, and 26 W m^{-2} for 30 to 180 min and, in Expt. 4, they were exposed at 107 W m^{-2} for 30–300 min each day. The data are means (n = 4; 10–133 eggs per replication), and the bars represent the standard errors. Means annotated with different letters are significantly different from each other according to Duncan's MRT tests at p = 0.05.

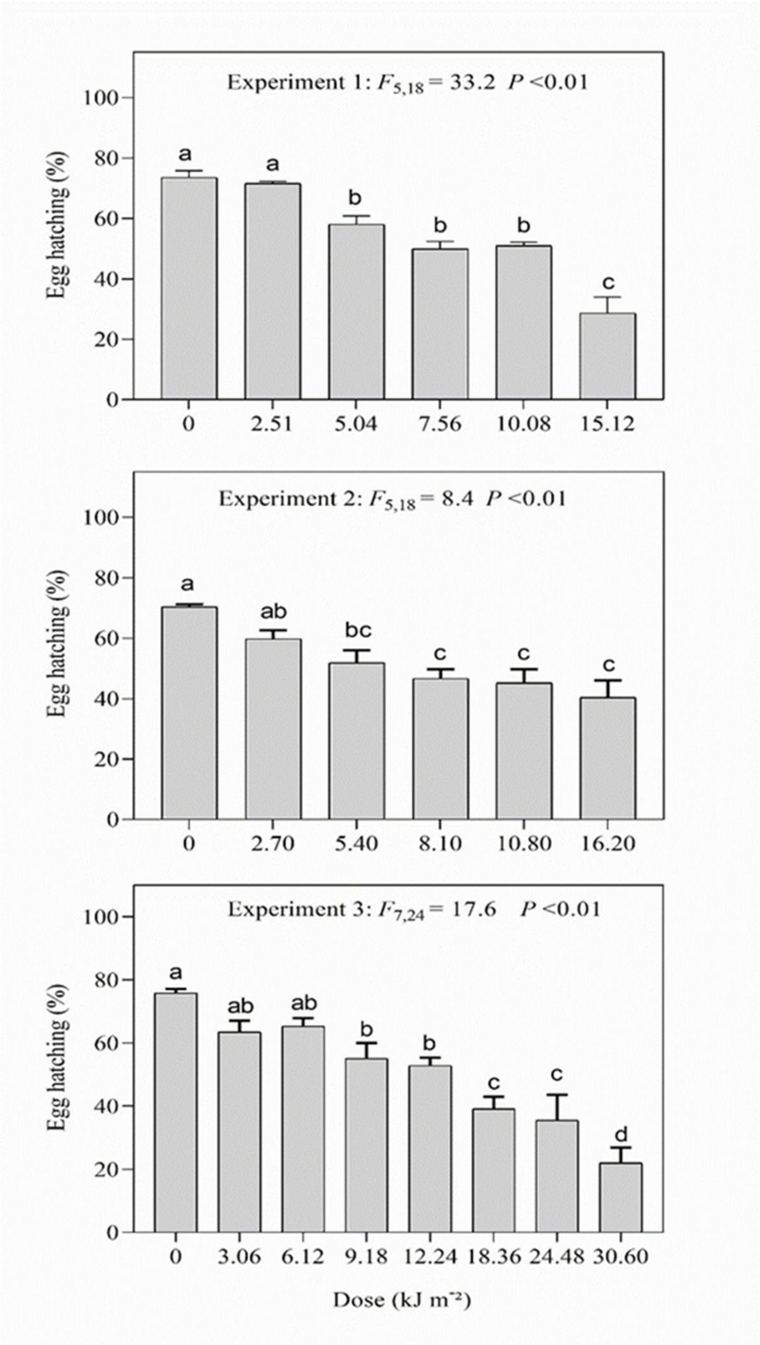

Figure S3. Effects of UV-B on egg hatching of *D. citri* following different exposure durations and intensities. In Expts. 1 and 2, the eggs were exposed daily to 1.4 and 1.5 W m^{-2} for 30 to 180 min and, in Expt. 3, they were exposed at 1.7 W m^{-2} for 30–300 min each day. The data are means (n = 4; 20–100 eggs per replication), and the bars represent the standard errors. Means annotated with different letters are significantly different from each other according to Duncan's MRT tests at p = 0.05.

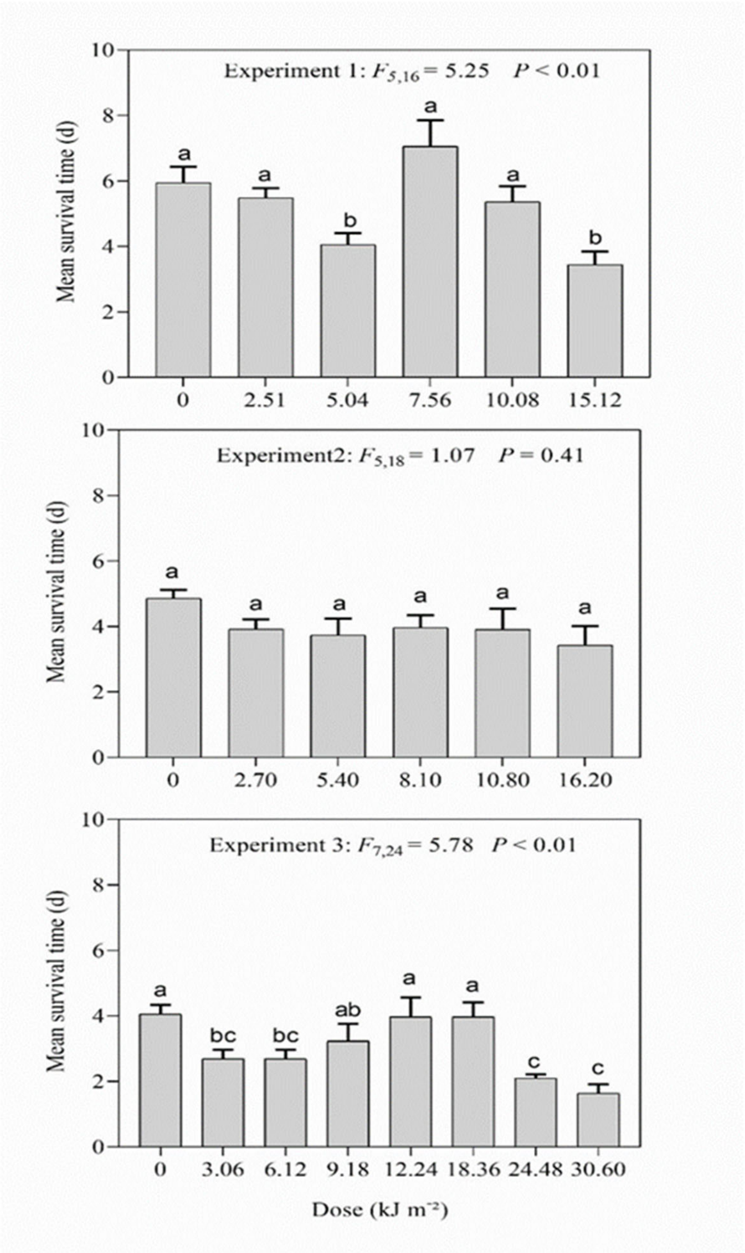

Figure S4. Effects of UV-B on survival of early nymphs that hatched from irradiated eggs. In Expts. 1 and 2, the eggs were exposed daily to 1.4 and 1.5 W m^{-2} for 30 to 180 min and, in Expt. 3, they were exposed at 1.7 W m^{-2} for 30–300 min each day. The data are means (n = 4; 5–100 nymphs per replication), and the bars represent the standard errors. Means annotated with different letters are significantly different from each other according to Duncan's MRT tests at $p = 0.05$.

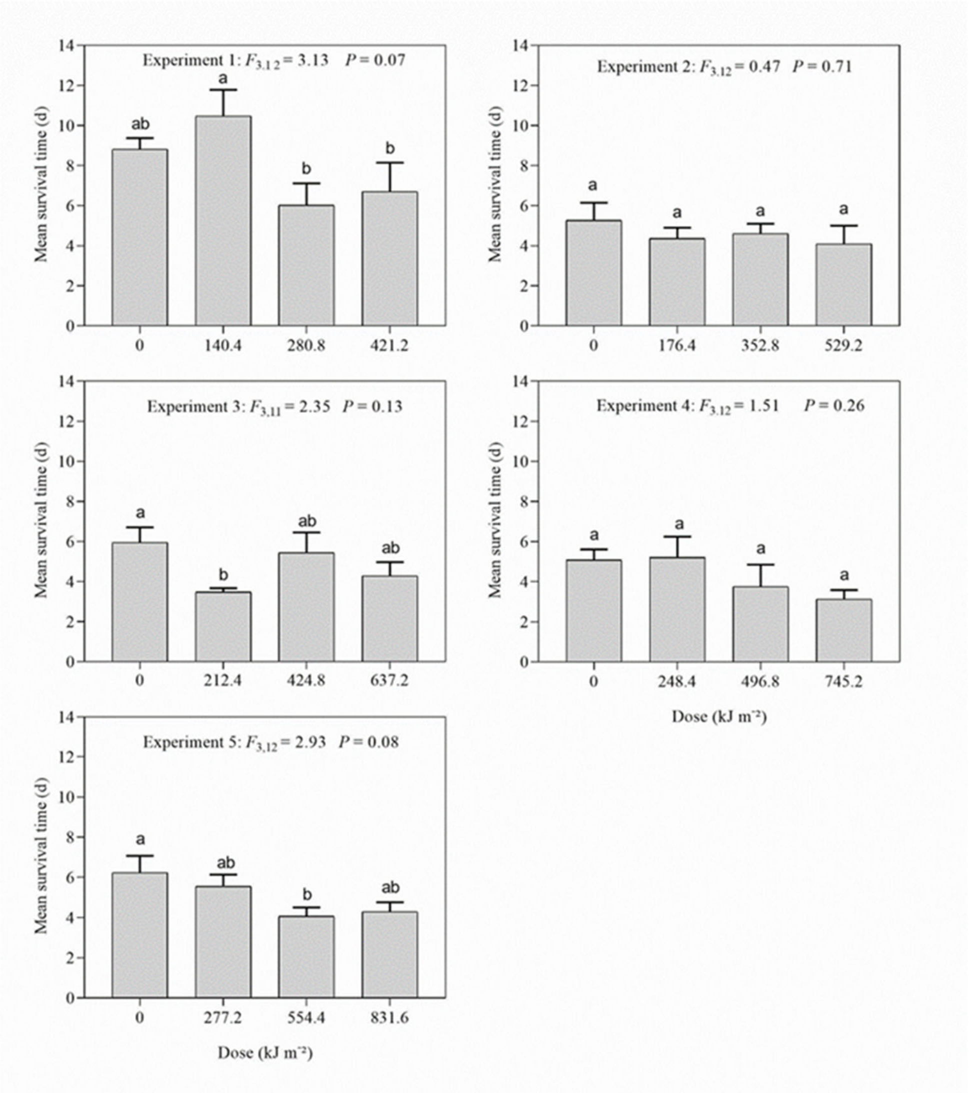

Figure S5. Effects of UV-A on survival of early instar nymphs of *D. citri* irradiated with UV-A. In Expts. 1, 2, 3, 4, and 5, the nymphs were, respectively, irradiated daily with 39, 49, 59, 69, and 77 W m^{-2} for 60 to 180 min. The data are means (n = 4; 20–100 nymphs per replication), and the bars represent the standard errors. Means annotated with different letters are significantly different from each other according to Duncan's MRT tests at p = 0.05.

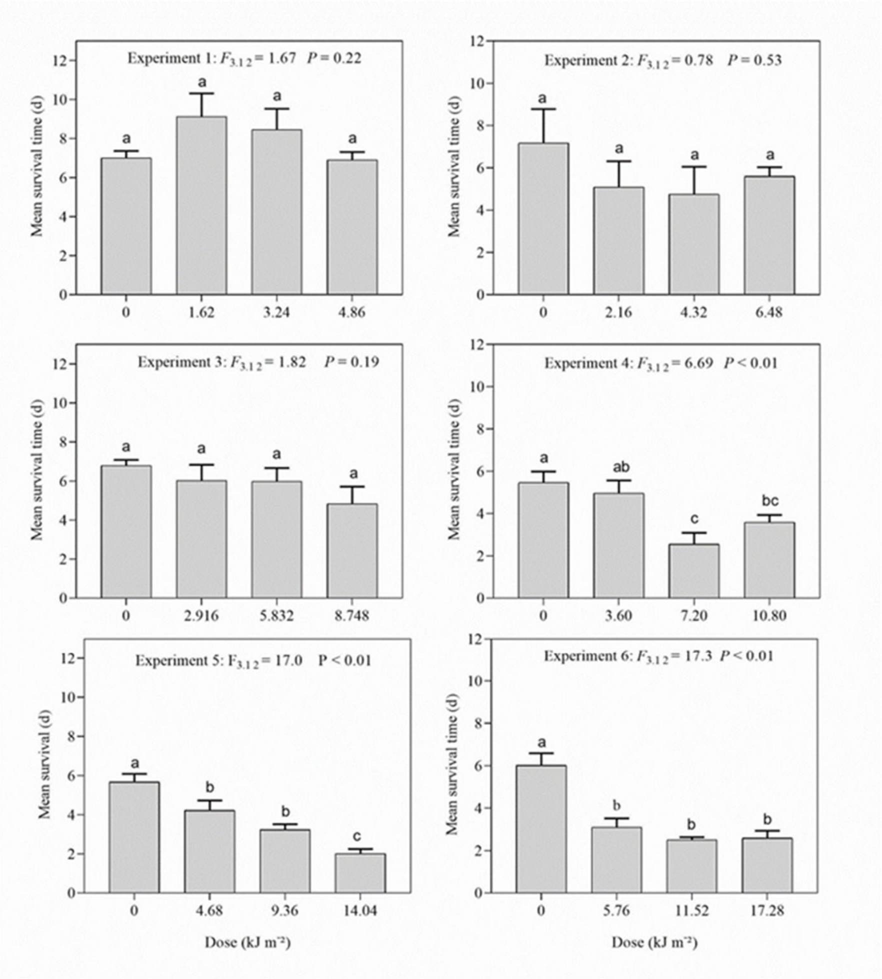

Figure S6. Effects of UV-B on survival of early instar nymphs of *D. citri* irradiated with UV-B. In the experiments the nymphs were irradiated daily at 0.45, 0.6, 0.81, 1, 1.3, and 1.6 W m^{-2} for 60 or 180 min. The data are means (n = 4; 20–100 nymphs per replication), and the bars represent the standard errors. Means annotated with different letters are significantly different from each other according to Duncan's MRT tests at p = 0.05.

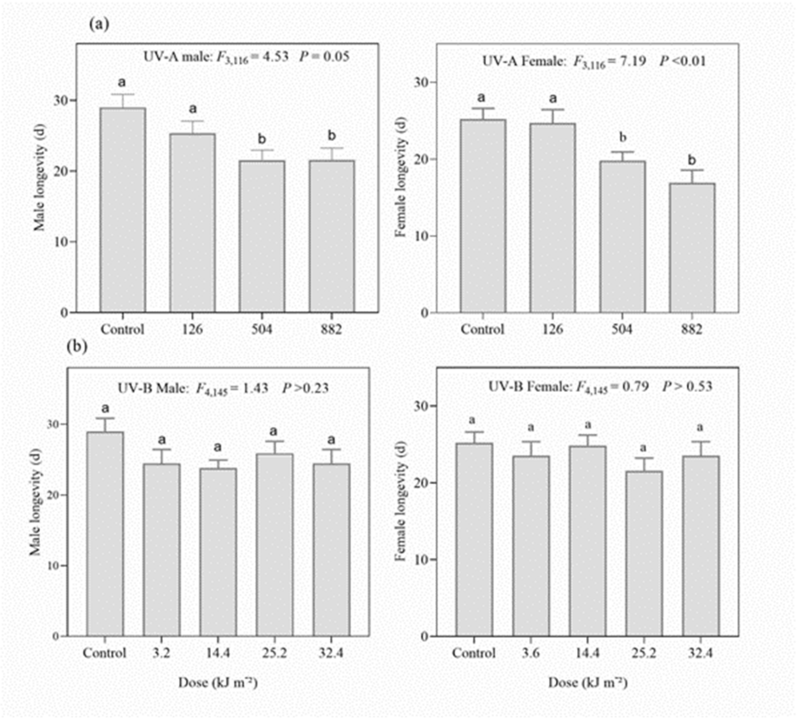

Figure S7. Effects of UV-A (a) and UV-B (b) on longevity of male and female adult *D. citri*. The adults were exposed daily to UV-A at 35 W m^{-2} for 1, 4, and 7 h or with UV-B at 1 W m−2 for 1, 4, 7, and 9 h each day until they died. The data are means (n = 30), and the bars represent the standard errors. Means annotated with different letters are significantly different from each other according to Duncan's MRT tests at p = 0.05.

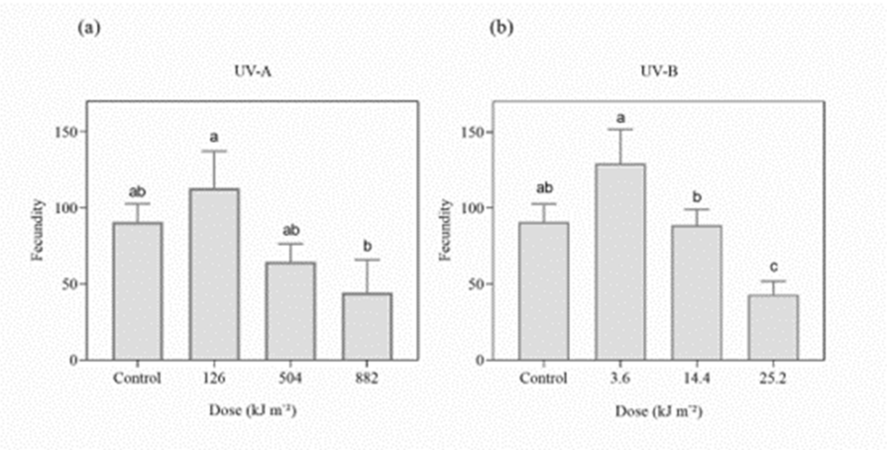

Figure S8. Effects of daily exposure to (a) UV-A at 35 W m^{-2} for 0, 1, 4, and 7 h (b) and UV-B at 1 W m^{-2} for 0, 1, 4, and 7 h on the fecundity of adult female *D. citri*; the insects were exposed until they died. The data are means (UV–A, $n = 6$–22; UV-B, $n = 7$–27), and the bars represent the standard errors. Means annotated with different letters are significantly different from each other according to Duncan's MRT tests at $p = 0.05$.

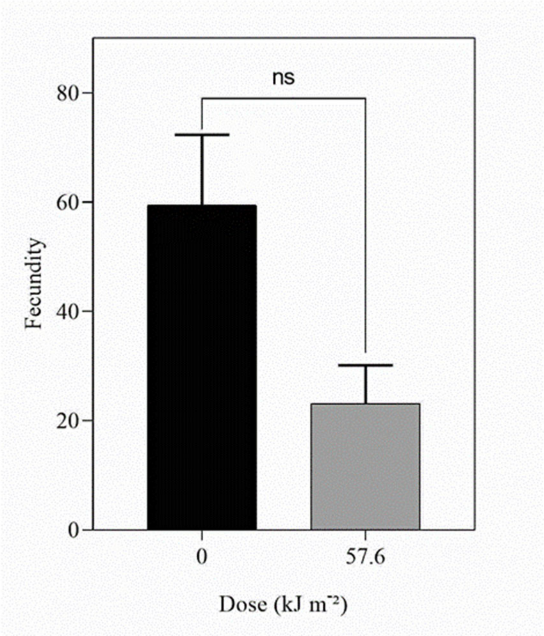

Figure S9. Effect of UV-B on the fecundity of female *D. citri* when treated for 16 h d^{-1}. The data are means ($n = 7$–19) and the bars represent the standard errors. Table S1. Effect of irradiation with UV-A on the F_1 generation. The data are means ($n = 6$–10) and the bars represent the standard errors.

Table S1. Effect of irradiation with UV-A on the F_1 generation. The data are means (n = 6–10), and the bars represent the standard errors.

Dose (kJ m^{-2} d^{-1})	Egg Hatch (%)	Nymphal Longevity * (Days)	Duration for Adult Eclosion (Days)	
			Male	Female
0	64.63 ± 2.66 a	5.50 ± 0.40 a	20.60 a	18.85 ab
126	63.33 ± 6.58 a	4.69 ± 0.31 a	19.50 a	21.25 a
504	66.52 ± 3.50 a	5.66 ± 0.60 a	19.28 a	17.00 b
882	19.66 ± 10.08 b			

Means annotated with different letters are significantly different from each other according to Duncan's MRT tests at $p = 0.05$. * Irrespective of instar.

Table S2. Effect of irradiation with UV-B on the F_1 generation. The data are means (n = 10), and the bars represent the standard errors.

Dose (kJ m^{-2} d^{-1})	Egg Hatch (%)	Nymphal Longevity * (Days)	Duration until Adult Eclosion (Days)	
			Male	Female
0	64.63 ± 2.66 ab	5.50 ± 0.40 ab	20.60 a	17.42 a
3.6	60.85 ± 1.30 ab	4.99 ± 0.36 b	20.50 a	19.00 a
14.4	69.47 ± 4.01 a	6.68 ± 0.80 a	20.67 a	20.33 a
25.2	60.03 ± 3.09 b	4.66 ± 0.22 b		

Means annotated with different letters are significantly different from each other according to Duncan's MRT tests at $p = 0.05$. *Irrespective of instar.
